# *Xylotrupes gideon* microchitosan-modified glass ionomer cement: *in vitro* assessment of mechanical properties

**DOI:** 10.3389/fdmed.2025.1717880

**Published:** 2025-12-18

**Authors:** Rosalina Tjandrawinata, Florencia Livia Kurniawan, Carolina Marpaung, Deviyanti Pratiwi, Eddy Eddy, Tansza Setiana Putri, Komariah Komariah, Indrayadi Gunardi, Sastra Kusuma Wijaya, Arief Cahyanto

**Affiliations:** 1 Department of Dental Materials, Faculty of Dentistry, Universitas Trisakti, Jakarta, Indonesia; 2 Department of Prosthodontics, Faculty of Dentistry, Universitas Trisakti, Jakarta, Indonesia; 3 Department of Oral Biology, Faculty of Dentistry, Universitas Trisakti, Jakarta, Indonesia; 4 Department of Oral Medicine, Faculty of Dentistry, Universitas Trisakti, Jakarta, Indonesia; 5 Department of Physics, Faculty of Mathematics and Sciences, Universitas Indonesia, Depok, Indonesia; 6 Department of Clinical Sciences, College of Dentistry, Ajman University, Ajman, United Arab Emirates; 7 Centre of Medical and Bio-allied Health Sciences Research, Ajman University, Ajman, United Arab Emirates

**Keywords:** mechanical properties, compressive strength, diametral tensile strength, glassionomer cement, hardness, microchitosan, *Xylotrupes gideon*

## Abstract

**Background:**

Glass ionomer cements (GIC) are valued as inherent fluoride-releasing dental restorative materials, while chitosan binds to negatively charged enamel surfaces, promoting mineral deposition and strengthening teeth. This study aimed to evaluate the mechanical properties of GIC modified with microchitosan derived from *Xylotrupes gideon*, using an *in vitro* experimental design. This study uniquely employs micro-scaled chitosan derived from the exoskeleton of Xylotrupes gideon, an insect-based, locally sourced, and environmentally sustainable alternative to conventional marine chitosan, to reinforce a conventional GIC.

**Materials and methods:**

Microchitosan was extracted from *Xylotrupes gideon* and incorporated into conventional GIC at 0.5%, 1% and 2% (w/w). Compressive strength, diametral tensile strength, and surface microhardness were measured using standard testing equipment after immersion in artificial saliva for 24 h and 7 days. Statistical analysis was performed using one-way ANOVA followed by the Games-Howell *post hoc* test, with significance set at *p* < 0.05.

**Results:**

The 1% microchitosan-modified GIC exhibited the most significant improvements compared to the unmodified control. After 7 days, compressive strength increased by 35.4%, diametral tensile strength by 51.3%, and surface hardness by 46.6% (*p* < 0.05). These enhancements are attributed to microscale reinforcement and chemical bonding between microchitosan and the GIC matrix.

**Conclusion:**

The addition of 1% microchitosan derived from *Xylotrupes gideon* significantly improved the mechanical performance of GIC. This bioactive reinforcement shows promising potential for clinical restorative applications, though further investigation into its long-term biocompatibility and fluoride release is warranted. These findings highlight a novel combination of insect-derived micro-scale chitosan and conventional GIC, yielding mechanical gains comparable to those reported for nanochitosan-modified formulations while relying on a more sustainable chitosan source.

## 1 Introduction

Dental composite materials, including glass ionomer cements (GICs), are widely recognized in restorative dentistry for their versatility, biocompatibility, and ability to bond chemically to dental hard tissues. GICs, in particular, are valued for their inherent fluoride release, which aids in the prevention of tooth decay and supports long-term tooth preservation (1, 2). These advantages make GICs ideal for pediatric dentistry and minimally invasive restorations where fluoride release and adhesion are critical; however, their adhesive performance varies significantly across formulations.

A major drawback of traditional GICs is their weak mechanical properties, including low compressive and tensile strength, poor wear resistance, and susceptibility to fracture under occlusal stress. These limitations preclude their use in stress-bearing restorations, particularly in posterior regions that are subjected to high occlusal loads (3). The other limitations are primarily attributed to the intrinsic chemistry of the GIC matrix, particularly the brittle nature of the glass phase and the sensitivity of the acid-base setting reaction to moisture and dehydration (4). Recent technology developments, particularly involving micro-sized chitosan and titanium dioxide, have demonstrated improved filler dispersion and particle–matrix bonding in glass ionomer cements, resulting in enhanced mechanical properties, matrix cohesion, fracture resistance, and bioactivity (5, 6) with chitosan enhancing particle-matrix bonding and decreasing microvoid formation (7).

Such reinforcement strategies yield structurally robust cement matrices characterized by minimized porosity and enhanced resistance to fracture propagation. Studies have shown that the incorporation of chitosan significantly enhances compressive and flexural strength, consistent with previous findings on quaternized chitosan-coated nanoparticles and carboxymethyl chitosan derivatives (8, 9) thereby improving matrix homogeneity and resistance to fracture propagation.

A previous study showed that the addition of chitosan to GIC has a positive effect on microhardness and improves surface roughness, making it a promising additive for dental restorative materials (10). This material reinforces the structural framework and enhances mechanical reliability, which in turn leads to improved fracture resistance, increased microhardness, and enhanced overall durability (11). The reinforcement effect demonstrated temporal dependence, with mechanical improvements more evident following extended maturation periods. Its ability to improve both the mechanical and biological properties of restorative materials makes it a compelling additive in dental biomaterials research. The molecular features of chitosan [poly-(b-1/4)-2-amino-2-deoxy-D-glucopyranose], a versatile biopolymer, are chemically inert, non-toxic, and possess robust, broad antimicrobial activities due to its polycationic nature (12). Characterized by amino and hydroxyl functional groups, chitosan enables ionic and hydrogen bonding that strengthens adhesion to dental substrates, improves mechanical strength through particle–matrix cohesion, and supports tissue compatibility via biocompatibility and regenerative potential (13) across a wide range of dental applications.

Chitosan exhibits remarkable physicochemical properties and good biocompatibility, forming bonds with human proteins, cells, and organs (14). Chitosan exhibits multifunctionality in dental applications by contributing to mechanical reinforcement, promoting tissue regeneration, and acting as a bioactive agent in restorative formulations such as glass ionomer cements and adhesives. Recently, alternative sources of chitosan have emerged, including the exoskeletons of the *Xylotrupes gideon* beetle, which offer a sustainable and locally available biomaterial. Previous research indicates that this chitosan, derived from insects, enhances GIC performance in acidic oral environments (with critical pH levels), showing promise for clinical applications (15). These advantages support further investigation of its mechanical reinforcement potential in dental composites. Research indicates that this novel form of chitosan demonstrates superior mechanical enhancement capabilities when integrated into GICs, including increased compressive strength and Vickers hardness (16). Additionally, the microstructure improves particle dispersion and matrix interaction, which are critical for achieving homogeneity and minimizing microvoid formation during the setting process (7, 17).

In addition to mechanical benefits, chitosan may confer secondary biofunctional advantages, which warrant future exploration but were not addressed in the present study (18). These properties may enhance the clinical performance of restorations by reducing bacterial colonization, improving marginal integrity, and supporting the healing of the pulp-dentin interface, in line with recent advancements in oral health technology (17). Notably, such modifications do not appear to compromise GIC's core biofunctional properties, such as fluoride ion release and adhesion to dentin (1, 11).

Therefore, this study aimed to investigate the effect of incorporating *Xylotrupes gideon*-derived microchitosan (on a submicron–micron scale) into conventional GIC on its mechanical properties, including compressive strength, diametral tensile strength, and surface hardness. By using an insect exoskeleton as a locally available and environmentally sustainable source of chitosan, this approach has potential for clinical feasibility, long-lasting nature, and biological functionality through the integration of eco-friendly materials in restorative dentistry (7).

## 2 Materials and methods

### 2.1 Synthesis and characterization of microchitosan from *xylotrupes gideon*

Microchitosan (MCH) was synthesized from the exoskeletons of *Xylotrupes gideon* beetles collected in Bogor, West Java, Indonesia. All body parts were detached and dried for 5 days, followed by extraction using established chitin-processing protocols: demineralization with 3N HCl, deproteinization with 3N NaOH, and deacetylation with 50% NaOH at 100°C to obtain chitosan.

**Figure 1:**
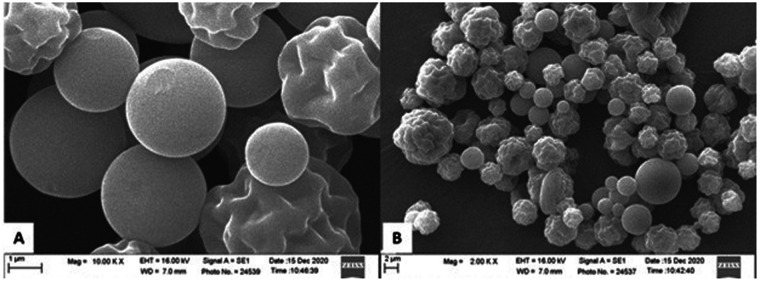
Scanning electron microscope images showing particles of microchitosan from *Xylotrupes gideon*. Image **(A)** highlights large, smooth spherical particles at 10,000 times magnification. Image **(B)** depicts a mixture of smaller, cougher textured and smooth spherical particles at 2,000 times magnification.

The resulting chitosan was converted into microform via ionic gelation with sodium tripolyphosphate (TPP) in deionized water, stirred at 2,500 rpm for 4 h, intentionally omitting acetic acid to improve biocompatibility. A particle size analyzer (Horiba, Japan) revealed a spherical morphology with a size distribution of 200–4,000 nm, while SEM analysis also confirmed the morphology of the chitosan particles, as shown in Figure 1. The synthesized MCH was stored at 4°C in airtight containers until use (19).

**Figure 2:**
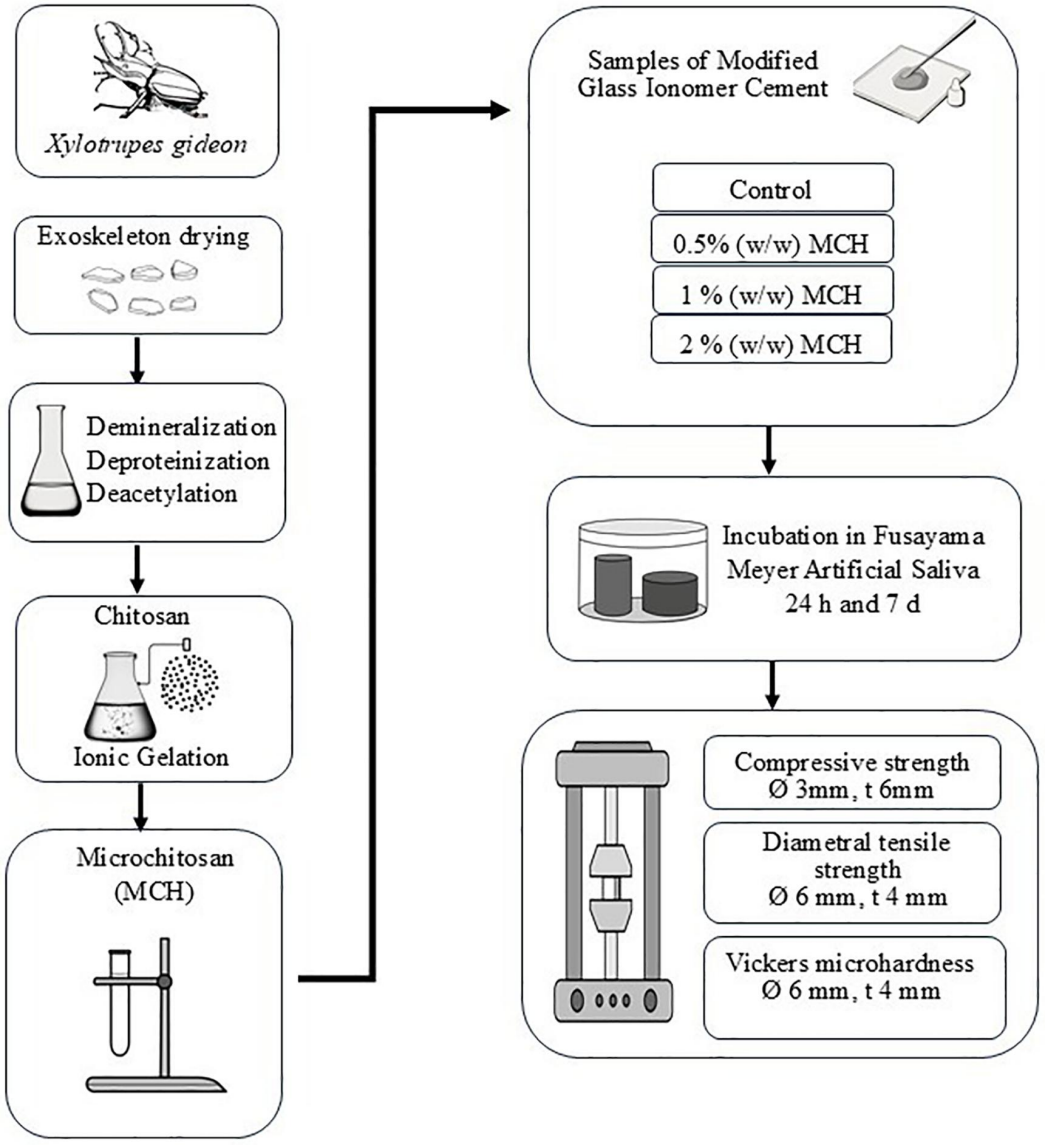
Flowchart illustrating the preparation and testing of modified glass ionomer cement (GIC). Starting with *Xylotrupes gideon* exoskeleton drying, it proceeds through demineralization, deproteinization, and deacetylation to obtain chitosan. Ionic gelation forms microchitosan (MCH). Samples of modified GIC are tested with 0.5%, 1%, and 2% (w/w) MCH addition. They undergo incubation in Fusayama Meyer artificial saliva for twenty-four hours and seven days. Tests include compressive strength, diametral tensile strength and Vickers microhardness, with specified diameters and thickness for each test.

The overall experimental workflow is illustrated in Figure 2, highlighting the synthesis of microchitosan from *Xylotrupes gideon* and its integration into glass ionomer cement for mechanical testing.

### 2.2 Preparation of modified glass ionomer cement

A commercially available Glass ionomer cement (Type IX Gold Label HS Posterior, GC, Tokyo, Japan) was used as the base material. The control group used unmodified GIC liquid, while the experimental groups consisted of 0.5%, 1%, and 2% (w/w) MCH added to the liquid component. Each modified liquid (0.5%, 1%, and 2% MCH) was vortex-mixed using the same standardized protocol immediately before mixing with the powder to improve homogeneity, acknowledging that achieving uniform dispersion becomes more challenging at higher MCH concentrations. Petroleum jelly was applied to the inner surfaces of the metal split mold. Then the mold was placed on a mylar strip, which was placed on a glass slab. The powder and liquid were dispensed on a paper pad and mixed manually according to the manufacturer's guidelines. After filling with the mixture, the mold was covered with another mylar strip, then covered with a second glass slab, and pressed for 30 s to extrude the excess material and obtain a uniformly smooth specimen surface. One hour after mixing, the specimens were removed from the mold, and any excess material was removed by dry grinding on both sides with 1,000-grit silicon carbide paper (1, 20, 21).

Compressive strength specimens were molded according to ISO 9917-1:2007: 3 mm diameter×6 mm height, while the specimens for diametrical tensile strength: 6 mm diameter × 4 mm thickness, and Vickers hardness: 6 mm diameter × 4 mm thickness. Specimen dimensions were confirmed using a digital caliper (Mitutoyo 500-196-30, Japan). After initial setting, all specimens were stored in 100% humidity at room temperature for 1 h. Compressive strength was calculated, in MPa, using the following equation: C = (4p)/(πd2) where p is the maximum force applied, in Newton; d is the average measured diameter of the specimen, in mm. The diametral tensile strength was also calculated in MPa, using the equation DTS = (2F)/(πdt), where F is the applied load, D is the diameter of the disk, and t is the thickness of the disk (22).

### 2.3 Conditioning and storage

A total of 80 specimens were prepared for each mechanical test and divided into four groups (*n* = 20 each), with each group further subdivided into two subgroups (*n* = 10) based on immersion time (24 h and 7 days). The minimum specimen size was estimated using the Lemeshow formula for comparing two independent means:

$$n=2\tau^{2}(Z_{1-\alpha }+Z_{1-\beta }{)}^{2}/(\mu_{1}-\mu_{2}{)}^{2}$$

Where *n* is the minimum number of specimens per subgroup, *μ₁* and *μ₂* are the expected means from a previous study, *τ* is the common standard deviation, Z_1−*α*_ is the Z value for the selected significance level, and Z_1−*β*_ is the Z value corresponding to the desired power.

A Fusayama-Meyer artificial saliva solution was prepared containing 0.4 g/L of NaCl, 0.4 g/L of KCl, 0.906 g/L of CaCl_2_ˑ2H_2_O, 0.690 g/L of Na_2_HPO_4_ˑ2H_2_O, 0.005 g/L of Na_2_Sˑ9H_2_O, and a pH of 6.75. Each specimen was immersed in 10 mL of artificial saliva in a sealed container and incubated at 37°C.

### 2.4 Mechanical testing

Mechanical properties were evaluated using a universal testing machine (Shimadzu, Japan) equipped with a 5 kN load cell, following validated protocols (23). Compressive and diametral tensile strength tests were conducted at a crosshead speed of 1 mm/min. Surface hardness was measured using a Shimadzu HMV-G microhardness tester (Kyoto, Japan), employing a micro Vickers diamond indenter with a 100 g load applied for 10 s (24). All mechanical testing adhered to ISO 9917-1:2007 guidelines and methodological frameworks.

### 2.5 Statistical analysis

All statistical analyses were performed using IBM SPSS Statistics 26.0 (IBM Corp., Armonk, NY, USA). Descriptive statistics, including the mean and standard deviation, were calculated. Data normality was assessed using the Kolmogorov–Smirnov test, and homogeneity of variances was evaluated using Levene's test. When the assumption of homogeneity was violated, one-way ANOVA was followed by the Games–Howell *post hoc* test for pairwise comparisons. Pearson correlation coefficients were calculated to examine relationships among mechanical parameters. The minimum required specimen size per subgroup was determined *a priori* using the Lemeshow equation for comparing two means, yielding a specimen size of 7. To ensure adequate power and consistency with ISO 9917-1:2007 recommendations and previous GIC mechanical studies, which typically use 5–10 specimens per subgroup, we ultimately used *n* = 10 specimens per subgroup. A significance level of *p* < 0.05 was applied throughout.

## 3 Results

**Figure 3:**
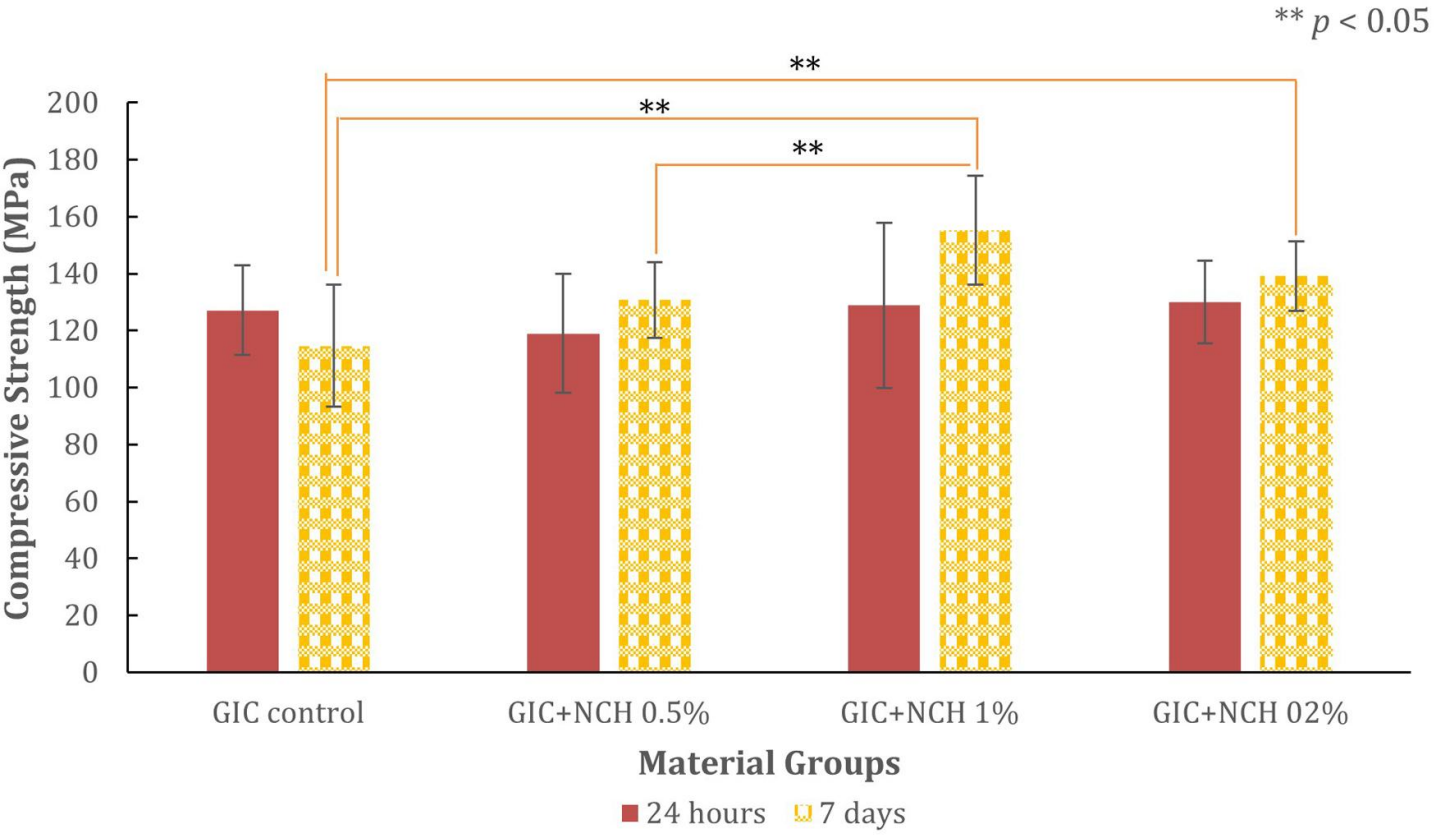
Bar chart showing compressive strength in MPa of GIC control and GIC with varying MCH percentages at 24 hours and 7 days. GIC + MCH 1% shows the highest strength. Significant difference, denoted by asterisks (**) indicate *p*<0.05 with ANOVA and *post hoc* Games-Howell.

**Figure 4:**
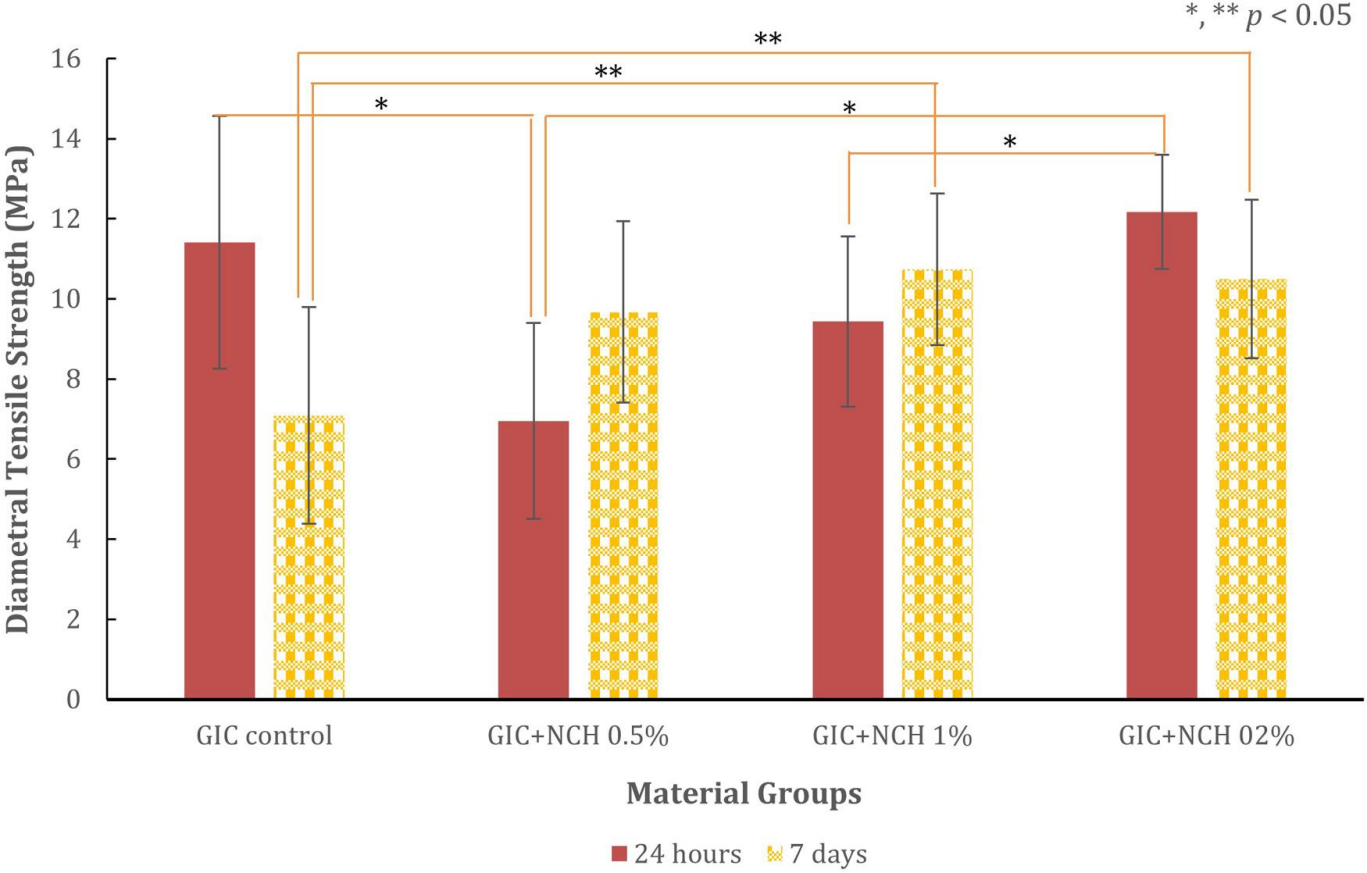
Bar chart showing diametral tensile strength (in MPa) for GIC control, GIC+MCH 0.5%, 1% and 2%. Strength measured at 24 hours (red bars) and 7 days (yellow patterned bars). Error bars indicate variability. Significant differences are marked with asterisks (*,**) for *p*-values less than 0.05 analyze with ANOVA and *post hoc* Games Howell.

**Figure 5:**
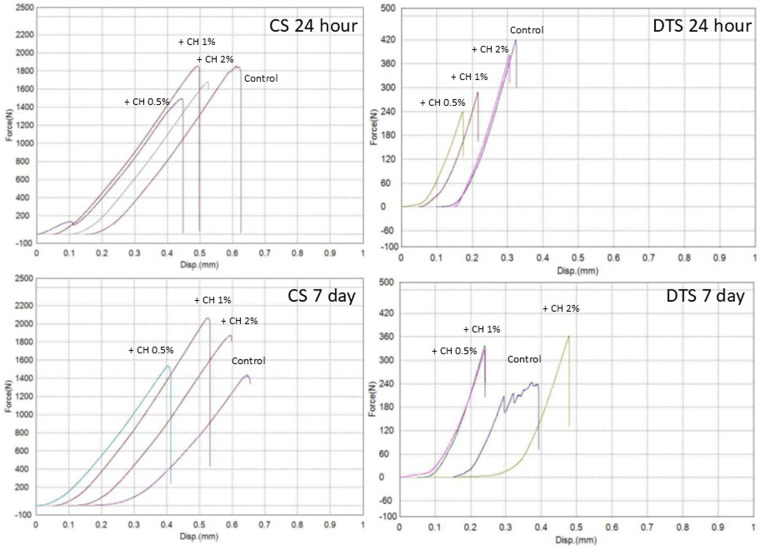
Four graphs compare the force versus displacement for different concentration of CH additives over time. All specimens were tested under monotonic loading to failure. Top left: CS 24 hour shows increasing force with higher CH concentrations. Top right: DTS 24 hour shows similar trends with lower force values. Bottom left: CS 7 day continues the pattern with increased force after seven days. Bottom right: DTS 7 day also shows the increase but with left force compared to CS graphs. The upward shift in the curves at 7 days, particularly in the 1% MCH group, reflects increased peak load and overall mechanical performance compared with the control.

**Figure 6:**
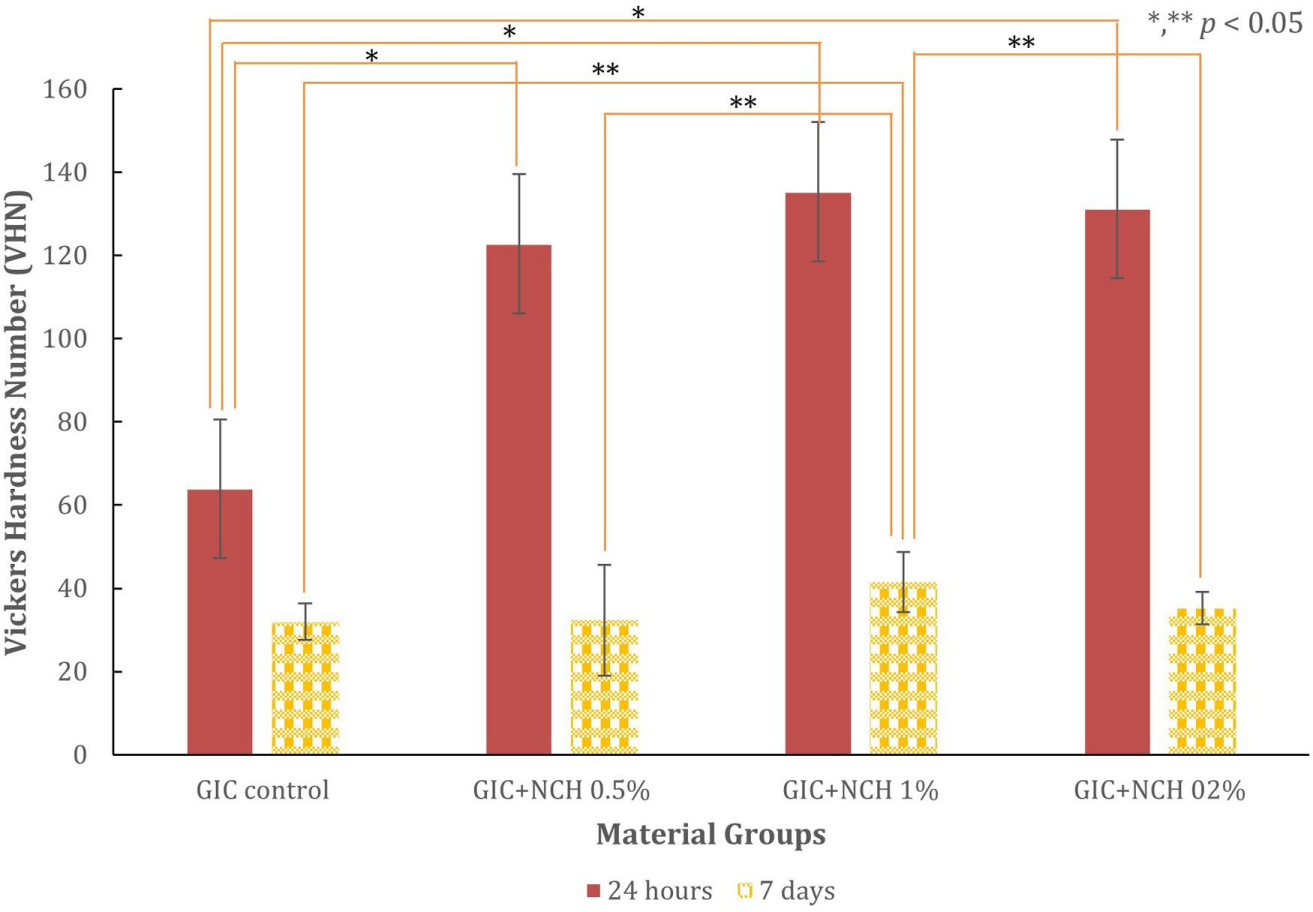
Bar chart comparing Vickers Hardness Number (VHN) across material groups: GIC Control, GIC + MCH 0,5%, GIC + MCH 1%, GIC + MCH 2%, measured at 24 hours and 7 days. Significant differences are indicated with asterisks for *p* < 0.05 using ANOVA and *post hoc* Games-Howell. The data clearly show that 1% microchitosan group exhibited the highest hardness values at both time points.

The compressive, diametral tensile strength, and hardness test results for all test groups are presented in Figures 3–6, while Table 1 presents the significance values for comparisons between groups. The data from 240 specimens (20 per group and 10 at different time points) were found to be normally distributed, as confirmed by the Kolmogorov–Smirnov test (*p* *=* *0.200*). The addition of microchitosan (MCH) at all concentrations resulted in statistically significant increases in both compressive and diametral tensile strength compared to the control group (*p* < 0.05).

**Table 1:** Games-Howell significant value (*p*) for 24 hours (white) and 7 days (grey) compressive strength, diametral tensile strength and vickers microhardness.

Compressive strength	Control	MCH 0.5%	MCH 1%	MCH 2%	Time
Control		0.223	0.002*	0.032*	7 days
MCH 0.5%	0.707		0.020*	0.473	
MCH 1%	0.671	0.234		0.155	
MCH 2%	0.973	0.476	0.848		
Time	24 hours				
Diametral tensile strength	Control	MCH 0.5%	MCH 1%	MCH 2%	Time
Control		0.133	0.014*	0.025*	7 days
MCH 0.5%	0.012*		0.674	0.825	
MCH 1%	0.445	0.118		0.992	
MCH 2%	0.900	<0.001*	0.040*		
Time	24 hours				
Vickers microhardness	Control	MCH 0.5%	MCH 1%	MCH 2%	Time
Control		1.000	<0.001*	0.341	7 days
MCH 0.5%	<0.001*		0.038*	0.913	
MCH 1%	<0.001*	0.719		0.003*	
MCH 2%	<0.001*	0.882	0.999		
Time	24 hours				

* Significant difference between groups with *p* < 0.05.

### 3.1 Compressive strength

The compressive strength results are presented in Figure 3. At 24 h, the control GIC group recorded a mean strength of 127.14 ± 15.82 MPa. Compared with the control, the 0.5% MCH group showed a slight reduction, whereas the 2% MCH group showed a numerically higher mean compressive strength (130.01 ± 14.40 MPa); however, this difference was not statistically significant (*p* = 0.973). The 1% MCH group was comparable to the control at this early time point.

After 7 days of immersion, the control group decreased to 114.63 ± 21.39 MPa. In contrast, all MCH groups showed higher values than the control. The highest compressive strength was observed in the 1% MCH group, which reached 155.16 ± 19.08 MPa (*p* = 0.002), representing a 35.4% increase over the 7-day control.

The corresponding compressive load-displacement curves (Figure 5, CS 24 h and CS 7 d) reveal predominantly linear elastic behavior, followed by abrupt failure in all groups, consistent with brittle, cement-like fracture. At 24 h, the slopes of the curves are broadly similar across groups, with only modest differences in peak load. After 7 days, the curves for all MCH groups shift towards higher peak loads compared with the control, with the 1% MCH group showing the highest peak load and largest displacement at failure, reflecting its superior strength and energy-absorbing capacity. The 0.5% MCH group exhibits the lowest peak load, while the 2% MCH and control curves lie between 0.5% and 1% MCH, mirroring the numerical ranking of compressive strength.

### 3.2 Diametral tensile strength

The diametral tensile strength results are presented in Figure 4. At 24 h, the control group showed a mean strength of 11.41 ± 3.16 MPa. Compared with the control, the 0.5% MCH group showed significantly lower strength, whereas the 1% and 2% MCH groups did not differ significantly from the control.

After 7 days, the control group decreased to 7.09 ± 2.70 MPa, while all MCH groups showed higher values. The 1% MCH group had the highest diametral tensile strength (10.73 ± 1.89 MPa, *p* = 0.014), corresponding to a 51% increase relative to the 7-day control.

The diametral tensile load-displacement curves (Figure 5, DTS 24 h and DTS 7 d) corroborate these trends. At 24 h, the control curve demonstrates the highest peak load, with the curves for the MCH groups clustered slightly below, reflecting the small or non-significant differences in DTS. By 7 days, the curves for all MCH-modified groups shift upward relative to the control, indicating higher peak loads at fracture. The 1% and 2% MCH groups in particular show steeper initial slopes and higher maxima than the control, whereas the 0.5% MCH curve occupies an intermediate position. This pattern is consistent with the 7-day DTS values, where 1% MCH provides the most pronounced reinforcement.

### 3.3 Vickers hardness

The Vickers microhardness test results at 24 h are given in Figure 6. The Control GIC had a microhardness mean value of 63.92 ± 15.17 VHN. The MCH-modified groups demonstrated significantly increased hardness, with a peak at 1% MCH (135.30 ± 17.90 VHN; *p* < 0.001).

All groups experienced a decrease in hardness after 7 days, while the 1% MCH maintained the highest residual value (46.95 ± 7.32 VHN, *p* < 0.001).

### 3.4 Trends and correlation analysis

The inclusion of microchitosan resulted in a consistent increase in mechanical properties, particularly at 1%. While the 2% group showed strong initial values, its performance declined slightly over time, suggesting an upper threshold of reinforcement.

Pearson correlation analysis revealed strong positive correlations between microchitosan concentration and compressive strength (r = 0.59, *p* = 0.002), diametral tensile strength (r = 0.57, *p* = 0.004), and hardness (r = 0.53, *p* = 0.011). Seven-day specimens of compressive strength and diametral tensile strength showed a positive correlation (r = 0.71, *p* = 0.001). Additionally, compressive strength and hardness were positively correlated (r = 0.64, *p* = 0.008), indicating an interconnected strengthening effect.

## 4 Discussion

### 4.1 Summary of findings

This study demonstrated that incorporating *Xylotrupes gideon*–derived microchitosan (MCH) into a conventional GIC significantly improved its mechanical properties. The 1% MCH concentration produced the best results across all measurements compressive strength, diametral tensile strength, and surface hardness, particularly after 7 days of immersion in artificial saliva, indicating a time-dependent reinforcement effect (25). The 0.5% MCH group showed only modest improvements, whereas the 2% MCH group, despite some initial enhancement, was likely more prone to particle agglomeration, which compromised its long-term stability (6).

These findings align with previous research showing that chitosan can enhance the strength and durability of GICs and fit within the broader trend of nanomaterial-reinforced dental composites (16). The superior performance of 1% MCH is consistent with reports that nanochitosan outperforms other nanostructures (e.g., nano-silica, nano-zirconia) in GICs, largely because of its ability to form hydrogen bonds with the polyacrylic acid matrix, thereby improving cohesion and ion exchange (1). Our results confirm substantial gains in surface hardness and compressive strength compared with both unmodified GIC and GICs containing other nano-additives (26), supporting the concept that small-particle chitosan is a promising modifier for stress-bearing restorations, especially in Class I and V cavities where fluoride release and biocompatibility are important (27).

All groups exhibited a decrease in hardness after 7 days, consistent with previous work reporting a 40%–45% decline in microhardness of conventional GICs following thermocycling (28, 29). Notably, the 1% MCH group retained the highest residual hardness (46.95 VHN), consistent with the notion that reinforced GICs resist degradation better than unmodified formulations.

### 4.2 Mechanical reinforcement

The mechanical enhancements observed with MCH-modified GIC can be explained by microscale dispersion and interfacial interactions between MCH and the GIC matrix. MCH particles (200–4,000 nm) can occupy microvoids and irregularities, similarly to the homogenizing effect reported for nano-hydroxyapatite/chitosan composites (30) and microhybrid composites, resulting in a denser, more uniform structure that reduces crack initiation and propagation. In addition, the amino and hydroxyl groups of chitosan form hydrogen and ionic bonds with polyacrylic acid chains, increasing cross-link density and internal cohesion (4, 17). In this way, microchitosan addresses microstructural weaknesses inherent to the acid–base setting mechanism and may support greater restoration durability (4).

At early maturation (24 h), however, MCH particles and their positively charged amino groups may interact competitively with polyacrylic acid and multivalent cations (Ca^2+^/Al^3+^), temporarily disturbing optimal ionic cross-linking within the GIC matrix. This competition, together with the introduction of additional interfaces, may reduce initial network rigidity and create localized microvoids, which is consistent with the modest reductions or limited improvements in compressive and diametral tensile strength observed at 24 h for some concentrations. With extended maturation (7 days) in artificial saliva, ongoing ion exchange and secondary cross-linking between the GIC matrix and microchitosan (via ionic and hydrogen bonding) likely produce a more cohesive, denser matrix, which explains the substantial strength gains at 7 days, particularly for the 1% group.

Beyond reporting the overall particle size range of 200–4,000 nm, more detailed particle size descriptors, such as the mean particle size, full size distribution profile, and polydispersity index, were not determined in this study. This represents a limitation of the current work, as such parameters would enable a more precise interpretation of how particle size, dispersion quality, and potential agglomeration contribute to the mechanical response of the different MCH concentrations. Future investigations will therefore include comprehensive physicochemical characterization of *Xylotrupes gideon*–derived microchitosan, including mean size, polydispersity, and surface charge, to better correlate these properties with the observed mechanical reinforcement of the GIC matrix.

The 1% concentration aligns with findings from previous studies, which have shown that 1%–1.5% chitosan maximizes mechanical gains in GICs without agglomeration, underscoring the critical role of nanoparticle concentration in dental composites (27). The good performance of 1% MCH is consistent with the general role of colloidal stability in such systems. Although zeta potential was not measured here, previous studies have shown that a high surface charge in chitosan-modified GICs improves nanoparticle dispersion, mechanical strength, and fluoride release (25). In contrast, quaternized chitosan-coated nanoparticles have demonstrated superior interfacial bonding and more uniform filler distribution, supporting improved flexural strength and fracture resistance in reinforced GIC systems (8). Together with reports on other nano-enhanced restorative formulations (3), these findings suggest that colloidal stability is a key determinant of functional integration and mechanical performance.

Our findings on the increase in Vickers surface hardness with MCH addition are in agreement with earlier studies, which show that chitosan positively influences GIC microhardness (10). Previous work comparing chitosan with hydroxyapatite revealed that chitosan not only preserved microhardness but was particularly effective in reducing surface roughness (31), supporting our conclusion that incorporating micro-sized chitin derivatives from *Xylotrupes gideon* contributes to a more durable and homogeneous GIC matrix. Furthermore, the incorporation of carboxymethyl chitosan into the liquid phase of GIC has been shown to significantly enhance flexural strength, fracture toughness, and compressive strength at low concentrations, reinforcing the view that chitosan derivatives strengthen the GIC network via hydrogen bonding and improved interfacial cohesion (9).

The decline in mechanical performance observed at 2% MCH is consistent with the tendency of higher nanoparticle concentrations to promote agglomeration and disrupt uniform dispersion (17). Conversely, very low concentrations (e.g., below 0.5%) may be insufficient to achieve effective reinforcement. In the present work, the interpretation that 2% MCH leads to increased agglomeration is therefore inferred from the mechanical trends and the known behavior of over-loaded particle-reinforced systems, rather than from direct microstructural evidence. Enhanced powder–liquid interactions during setting, driven by the high surface energy of MCH, likely contributed to favorable gelation and final structure at 1%, in line with observations that optimized powder–liquid interactions support improved mechanical outcomes (32). MCH thus appears to improve hardness and mechanical durability while preserving essential GIC properties, including adhesion (33). At the same time, the absence of fractographic SEM analysis of fractured GIC–MCH specimens in this study is a further limitation that prevents direct visualization of crack paths, particle distribution, and agglomerate formation at different MCH concentrations. A dedicated follow-up investigation, including fracture-surface SEM for all MCH loadings is planned to elucidate these microstructural–mechanical correlations more clearly.

Previous work has shown that resin coatings can significantly enhance the viscoelastic behavior and environmental resistance of highly viscous GICs (34). While such strategies focus on external surface protection, our results suggest that internal reinforcement with chitosan may offer comparable benefits by increasing matrix cohesion and long-term mechanical stability without necessitating additional coating layers. In another study, shrimp shell–derived chitosan combined with hydrophilic fibers yielded a composite with compressive strength of 32.67 MPa and tensile strength of 17.18 MPa (35). Although their tensile strength values were higher, our 1% MCH–GIC formulation achieved a much higher compressive strength (155.16 MPa),

When compared with other reinforced GICs, the 1% MCH-GIC formulation (compressive strength 155.16 MPa) approaches the values reported for zirconia-reinforced GIC (160.91 MPa) and silver-alloy GIC (151.47 MPa) (21). These results exceed the ISO 9917-1:2016 minimum compressive strength threshold of 100 MPa for conventional restorative GICs (36). Although the compressive strength remains below that of enamel (262 MPa) and dentin (234 MPa), and below values for some high-viscosity GICs (301 MPa), it is still well above the estimated stress generated by maximum bite forces (≈21.2 MPa, assuming a 20 mm^2^ contact area) (22, 37). Thus, the present MCH-GIC appears mechanically adequate for typical masticatory loading, and further optimization, such as combining MCH with high-viscosity GIC may provide additional safety margins.

In addition to bulk mechanical strengthening, chitosan has been reported to enhance the shear bond strength of GIC to dentin (38), which is critical for long-term clinical success. While bonding behavior was not evaluated in this study, it is plausible that the microchitosan used could confer similar benefits; this will be addressed in future work.

### 4.3 Biofunctional attributes and fluoride release

Biofunctional attributes, including antimicrobial potential, remineralization support, and tissue compatibility, are highly relevant to restorative materials. The present study did not evaluate antibacterial activity, fluoride release, or pulp/tissue responses, and no biological claims are made based on our data. However, previous studies have shown that chitosan's positively charged surface can disrupt bacterial membranes and inhibit biofilm formation (18, 39, 40), and that *Xylotrupes gideon*–derived nanochitosan can exhibit antibacterial effects and support sustained fluoride release in experimental restorative formulations (19, 41).

Notably, prior work indicates that incorporating nanochitosan into GIC does not compromise fluoride release and may even enhance it, particularly under acidic conditions (16, 25, 42). Chitosan's hydrogel-like network may modulate ion diffusion and support prolonged fluoride availability. Other reports have demonstrated that chitosan can promote the proliferation of fibroblasts and odontoblasts, enhance pulp healing, and reduce inflammation when applied near pulp tissue (43, 44). MCH's mucoadhesive behavior may also improve marginal sealing *in vivo* (45). Together, these findings suggest that chitosan-modified GICs could offer multifunctional benefits, combining mechanical reinforcement with potential antibacterial and remineralizing effects; nevertheless, such biofunctional properties remain to be tested specifically for the present MCH-GIC formulation and should be addressed in future *in vitro* and *in vivo* studies.

### 4.4 Clinical implications and future directions

Within the limitations of this *in vitro* study, *Xylotrupes gideon*–derived microchitosan appears to be a promising internal reinforcement for conventional GIC, particularly at a 1% loading. The observed improvements in strength and hardness, together with the material's fluoride-releasing nature, support potential use in stress-bearing posterior restorations, atraumatic restorative treatment (ART), and liners or bases in deep cavities, provided that adequate adhesion and long-term behavior are confirmed (1, 16, 27). However, the absence of flexural strength data represents an important limitation, as flexural performance is highly relevant to the clinical loading conditions of GIC restorations. To address this, future work will incorporate three-point or biaxial flexural strength testing as part of a broader mechanical characterization of *Xylotrupes gideon*-derived microchitosan–modified GICs.

Further work is required before clinical adoption. Priority areas include: (i) *in vivo* assessment of pulp and tissue responses, (ii) long-term durability under occlusal load, thermocycling, and pH cycling, (iii) evaluation of cytotoxicity and potential allergic reactions, particularly in pediatric and medically compromised patients, (iv) detailed studies of fluoride-release kinetics under cariogenic challenges, and (v) optimization of handling and setting characteristics for clinical workflow.

Finally, the use of *Xylotrupes gideon*–derived microchitosan highlights an environmentally conscious approach to dental biomaterials. Insect-based chitosan offers a locally available, potentially less allergenic alternative to marine sources and integrates effectively into GIC matrices (1, 16, 18). Microchitosan thus represents a sustainable modifier that can enhance the mechanical performance of GIC while reducing reliance on traditional marine-derived additives. Such improvements in mechanical properties are critical for the biomechanical performance of GIC restorations in the oral cavity. Comprehensive biocompatibility testing, biofunctional evaluation, and clinical trials are necessary to confirm the safety and effectiveness of this approach and to support its broader clinical implementation.

## 5 Conclusion

Incorporating 1% microchitosan (MCH) from *Xylotrupes gideon* into glass ionomer cement (GIC) significantly enhanced its mechanical properties after 7 days of maturation in artificial saliva. This modification resulted in a 35% increase in compressive strength, a 51% improvement in tensile strength, and 46% increase in surface hardness. Although the 2% MCH group initially showed strong values, its performance declined over time, suggesting an upper threshold of reinforcement. This reduced performance was likely due to particle agglomeration, which undermined long-term stability. A 0.5% formulation produced inconclusive results, the optimized 1% formulation supports minimally invasive dentistry by prolonging restoration longevity and preserving tooth structure.

## Data Availability

The raw data supporting the conclusions of this article will be made available by the authors, without undue reservation.
